# The challenges of identifying and studying type 1 diabetes in adults

**DOI:** 10.1007/s00125-023-06004-4

**Published:** 2023-09-20

**Authors:** Nicholas J. Thomas, Angus G. Jones

**Affiliations:** 1 Department of Clinical and Biological Sciences, University of Exeter, Exeter, UK; 2 Royal Devon University Healthcare NHS Foundation Trust, Exeter, UK

**Keywords:** Age, C-peptide, Genotype, Islet autoantibodies, Phenotype, Prior probability, Review, Type 1 diabetes adult-onset

## Abstract

Diagnosing type 1 diabetes in adults is difficult since type 2 diabetes is the predominant diabetes type, particularly with an older age of onset (approximately >30 years). Misclassification of type 1 diabetes in adults is therefore common and will impact both individual patient management and the reported features of clinically classified cohorts. In this article, we discuss the challenges associated with correctly identifying adult-onset type 1 diabetes and the implications of these challenges for clinical practice and research. We discuss how many of the reported differences in the characteristics of autoimmune/type 1 diabetes with increasing age of diagnosis are likely explained by the inadvertent study of mixed populations with and without autoimmune aetiology diabetes. We show that when type 1 diabetes is defined by high-specificity methods, clinical presentation, islet-autoantibody positivity, genetic predisposition and progression of C-peptide loss remain broadly similar and severe at all ages and are unaffected by onset age within adults. Recent clinical guidance recommends routine islet-autoantibody testing when type 1 diabetes is clinically suspected or in the context of rapid progression to insulin therapy after a diagnosis of type 2 diabetes. In this moderate or high prior-probability setting, a positive islet-autoantibody test will usually confirm autoimmune aetiology (type 1 diabetes). We argue that islet-autoantibody testing of those with apparent type 2 diabetes should not be routinely undertaken as, in this low prior-prevalence setting, the positive predictive value of a single-positive islet antibody for autoimmune aetiology diabetes will be modest. When studying diabetes, extremely high-specificity approaches are needed to identify autoimmune diabetes in adults, with the optimal approach depending on the research question. We believe that until these recommendations are widely adopted by researchers, the true phenotype of late-onset type 1 diabetes will remain largely misunderstood.

**Graphical Abstract:**

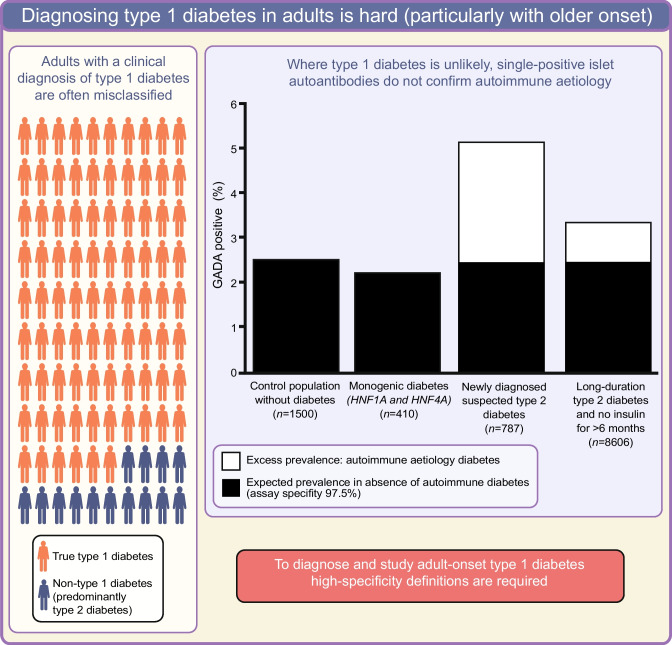

**Supplementary Information:**

The online version contains supplementary material, including a slideset of the figures for download, available at 10.1007/s00125-023-06004-4.


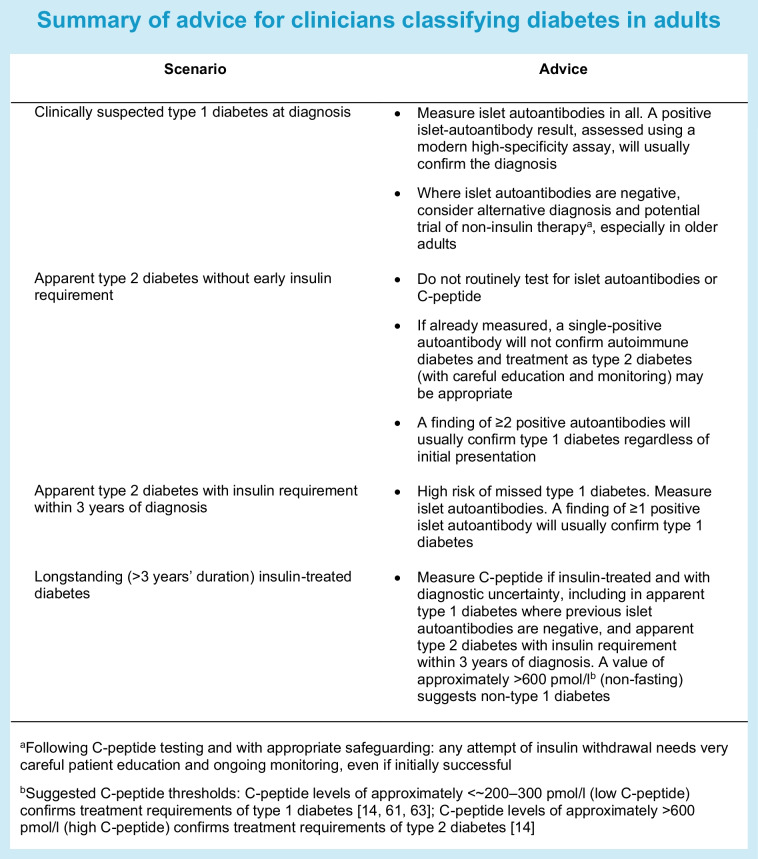


## Introduction

Type 1 diabetes has long been regarded as a disease predominantly of childhood and adolescence. While recognised that type 1 diabetes occurs at any age, only recently has it been appreciated that over half of type 1 diabetes cases occur in adulthood [1–3]. Despite this, the phenotype of type 1 diabetes that presents in adults, especially in older adults, is poorly understood.

Type 1 diabetes that develops in adults is frequently reported to have a different phenotype from the disease occurring in children and young adults. One potential reason for many of the reported differences in phenotype with onset age is the difficulty in identifying type 1 diabetes in adults, in whom type 2 diabetes predominates. The impact of low prior prevalence (i.e. type 1 diabetes being rare in comparison with type 2 diabetes in those with new-onset diabetes) means that, in isolation, clinical diagnosis or single-positive islet autoantibodies have insufficient specificity for robust diagnosis [4–7]. Therefore, adult type 1 diabetes cohorts defined by clinical diagnosis or islet autoantibodies alone are likely to include non-autoimmune diabetes cases, resulting in a mixed, more type 2 diabetes-like phenotype when compared with type 1 diabetes presenting in children, in whom type 1 diabetes predominates.

This article aims to review the current literature, highlighting the challenges of diagnosing type 1 diabetes in adults. We explore how difficulty in classification has an impact on our understanding of the phenotype of adult-onset type 1 diabetes, and how classification might be improved in both clinical practice and research.

## Adult-onset type 1 diabetes is difficult to diagnose and misclassification is common

### In adults with diabetes, type 2 diabetes predominates over type 1 diabetes

**Fig. 1:**
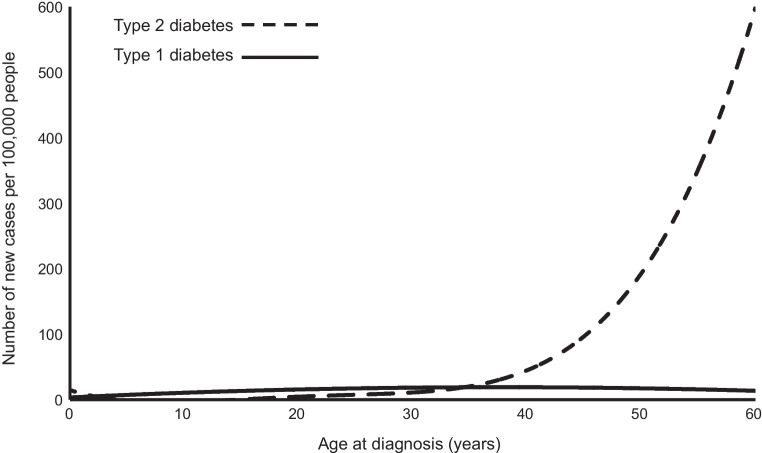
The incidence of genetically defined type 1 and type 2 diabetes in the first six decades of life. Recreated from original data from UK Biobank [1]. This figure is available as part of a downloadable slideset

Much of what is known about type 1 diabetes comes from studying children, where the majority of those who develop diabetes have type 1 diabetes (85–95%). In children, type 2 diabetes is usually associated with severe obesity and monogenetic diabetes (MODY) is rare (1–3%) [8–10], meaning almost all of those diagnosed with type 1 diabetes are correctly classified. Conversely, in adults >95% of incident diabetes cases are type 2 diabetes and presentation of type 1 diabetes and type 2 diabetes substantially overlap, making type 1 diabetes identification difficult, particularly with increasing onset age, which is strongly associated with increasing type 2 diabetes risk [1]. Figure 1 shows that the development of genetically defined type 1 diabetes is consistent across the first six decades of life, but that type 2 diabetes is significantly more common as age increases [1, 2]. This appears true worldwide, although data from low- and middle-income countries is limited [3].

### No clinical definition of type 1 diabetes exists

Current type 1 diabetes definitions are based on pathophysiology. The ADA defines type 1 diabetes as diabetes due to autoimmune beta cell destruction, usually leading to absolute insulin deficiency, or severe insulin deficiency without evidence of beta cell autoimmunity (idiopathic type 1 diabetes) [11]. The World Health Organization defines type 1 diabetes as beta cell destruction leading to absolute insulin deficiency [12]. Capturing these pathophysiological definitions within clinical practice can be difficult. In most settings, type 1 diabetes is clinically diagnosed based on routinely available clinical features. While the presence of islet autoantibodies supports a diagnosis and allows direct assessment of aetiology, tests for autoantibodies are not routinely conducted or always available, and these have imperfect sensitivity and specificity [4, 13]. While the treatment requirements of type 1 diabetes appear to be principally mediated through development of severe insulin deficiency, which is measurable using C-peptide, insulin secretion can be maintained at type 1 diabetes diagnosis and levels overlap with other diabetes types. Therefore, for diabetes classification, C-peptide testing has the greatest utility in long-duration disease [14].

### In adults, no single feature confirms type 1 diabetes

Clinical features of type 1 diabetes, type 2 diabetes and other diabetes subtypes substantially overlap and no single feature confirms type 1 diabetes [6]. In adults, developing type 2 diabetes at low body weight is not uncommon, and both ketoacidosis and profound presentation hyperglycaemia occur in non-autoimmune, apparent type 2 diabetes, without requirement for long-term insulin treatment [15, 16]. In the UK, 7% of individuals with apparent type 2 diabetes who are well controlled with metformin, have a BMI <25 kg/m^2^ [7]. In the USA, 10% of young individuals with apparent type 2 diabetes had ketoacidosis as a presenting feature [17], and 60% of adults within a multi-ethnic population presenting with ketoacidosis had evidence of preserved C-peptide [18].

While a relatively small proportion of individuals with apparent type 2 diabetes present with severe symptoms, because type 2 diabetes is many times more common than type 1 diabetes in adults, the majority of those with severe symptoms may have type 2 diabetes. In other words, an atypical presentation of type 2 diabetes may be more common than a ‘classical’ presentation of type 1 diabetes. Therefore, differentiating these atypical cases from type 1 diabetes is extremely challenging. The influence of prior prevalence (Bayes’ theorem) and the sensitivity and specificity of a biomarker test/clinical feature on the positive predictive value (PPV) of the test/feature is shown mathematically in the following equation:

$$\mathrm{PPV}=\frac{\mathrm{sensitivity }\times \mathrm{ prevalence}}{(\mathrm{sensitivity }\times \mathrm{ prevalence})+(\left(1-\mathrm{specificity}\right) \times \left(1-\mathrm{prevalence}\right))}$$

Table 1 uses the above equation to illustrate how low prior prevalence of type 1 diabetes in adults means that even features with high specificity may have a modest predictive value for the disease. These issues will be more marked in populations and ethnicities associated with increased type 2 diabetes risk, and/or with the occurrence of type 2 diabetes at younger age and lower BMI [19, 20]. For BMI, this is further exacerbated by the inverse relationship between onset age and BMI [21].

**Table 1:** Illustrative example of the impact of prevalence on PPV of clinical features for type 1 diabetes

Clinical feature	Sensitivity^a^	Specificity^a^	PPV in diabetes diagnosed at <20 years of age^b^ (96% T1D)^c^	PPV in diabetes diagnosed at ≥50 years of age^b^ (1% T1D)^c^
BMI <25 kg/m^2^	55%	90%	99%	5%
Weight loss	81%	72%	99%	3%
HbA_1c_ >115 mmol/mol (12.7%)	33%	87%	98%	3%
Glucose >20 mmol/l	38%	85%	98%	2%
Lack of diabetes family history^d^	66%	64%	98%	2%
Osmotic symptoms	94%	32%	97%	1%
Other autoimmune disease	15%	93%	98%	2%

^a^Sensitivity and specificity derived from data for participants with recently diagnosed (median duration 5 months) adult-onset diabetes in the StartRight Study [37], and relate to performance of the feature (and threshold) stated for type 1 diabetes, which was defined by ≥2 positive islet autoantibodies (of GAD, IA-2 or ZnT8) or C-peptide <200 pmol/l (vs type 2 diabetes, which is defined by three negative islet autoantibodies and C-peptide >600 pmol/l)

^b^PPV calculated as follows: $\mathrm{PPV}=\frac{\mathrm{sensitivity }\times \mathrm{ prevalence}}{(\mathrm{sensitivity }\times \mathrm{ prevalence})+(\left(1-\mathrm{specificity}\right) \times \left(1-\mathrm{prevalence}\right))}$

Note in the above equation, sensitivity and specificity should be in the range of 0 to 1 (values in % should be divided by 100)

^c^Prior prevalence of type 1 diabetes at diagnosis based on incidence data from the Scottish Diabetes Survey 2020 [65]

^d^Parent or sibling with diabetes

T1D, type 1 diabetes

### Positive islet autoantibodies may not confirm autoimmune aetiology diabetes if the likelihood of the disease is low

Islet autoantibodies are strongly associated with type 1 diabetes, being found at diagnosis in ~90% of childhood cases [13, 22]. However, islet autoantibodies are detected in other forms of diabetes and in individuals without diabetes [23], a situation in common with autoantibodies for a wide range of autoimmune conditions [24]. Importantly, islet autoantibodies themselves are not thought to be the causative agent for autoimmune beta cell destruction, and their presence does not confirm cellular autoimmunity at the level of the islet [25, 26].

**Fig. 2:**
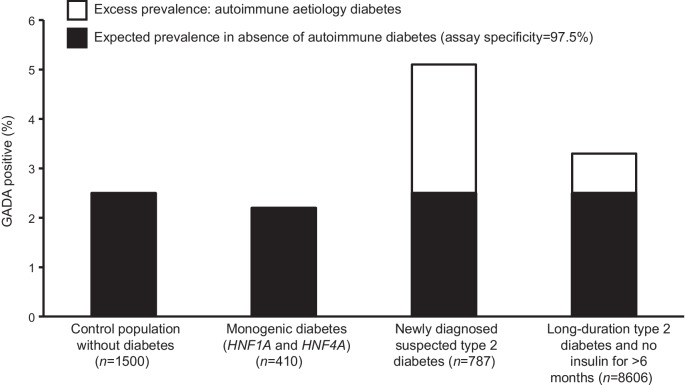
The prevalence of GADA positivity for an assay and threshold with 97.5% specificity (positive defined as >10 units/ml) for: (1) a control population without diabetes (HbA_1c_ <48 mmol/mol [<6.5%]) (data from [66]); (2) individuals with *HNF1A* and *HNF4A* MODY (T. J. McDonald, University of Exeter, UK, unpublished data associated with [67]); (3) individuals aged ≥18 years, with recently diagnosed diabetes that was initially treated as type 2 diabetes (without initial insulin for >2 weeks) (A. G. Jones, University of Exeter, UK, unpublished data from the StartRight Study [31, 37]); and (4) individuals with long-duration type 2 diabetes, aged ≥35 years at diagnosis, with a clinical diagnosis of type 2 diabetes, absence of insulin requirement within 6 months of diagnosis and with median diabetes duration of 11 years (data from [6]). This figure is available as part of a downloadable slideset

The prevalence of islet autoantibodies in people with diabetes that is not autoimmune in origin will mirror the prevalence of islet autoantibodies in healthy control populations (calculated as 1−test specificity). This issue has an impact on the interpretation of islet-autoantibody results in adults, given that type 2 diabetes is substantially more common than type 1 diabetes (Fig. 1). This issue becomes more pronounced if the likelihood of autoimmune diabetes is further reduced by selecting individuals with a type 2 diabetes phenotype, as seen in the study of latent autoimmune diabetes of adults (LADA), and remains an important issue despite recent improvements in islet-autoantibody assays [4, 23, 27]. Figure 2 shows GADA prevalence using an assay and threshold with 97.5% specificity (derived from a large [*n*=1500] control population), in individuals with genetically confirmed MODY (*n*=410), individuals with recent-onset clinically suspected type 2 diabetes (*n*=787) and individuals with long-duration type 2 diabetes (*n*=8606). Consistent with the assay specificity of 97.5%, ~2.5% of those with monogenic diabetes (non-autoimmune aetiology) tested positive. In new-onset diabetes initially treated as type 2 diabetes, the prevalence of GADA using this assay and threshold was only modestly higher (5.1%) than the expected prevalence in cases without autoimmune disease (2.5%; Fig. 2). This suggests that while autoimmune diabetes is present, even with a high-specificity assay that reports near-perfect specificity in an international standardisation programme (reported specificity in the 2018 and 2020 international islet-autoantibody standardisation programme control cohort [*n*<100] was 100% and 98.9%, respectively [27]), the PPV of the test is modest; consistent with Bayes’ theorem a high proportion of islet-autoantibody-positive individuals will not have autoimmune aetiology diabetes. Importantly, testing for multiple autoantibodies would further reduce specificity and, therefore, PPV of a single-positive result. The intermediate phenotype of LADA may be explained by this issue, as previously discussed [4, 5]. As we have recently reviewed this explanation for the characteristics of LADA in detail, and this group is diagnosed clinically with type 2 diabetes, we have focused on conventional type 1 diabetes for the remainder of this article [4]

### In clinical practice, type 1 diabetes classification in adults is difficult and misclassification is common

In adult-onset diabetes, the scarcity of type 1 diabetes relative to type 2 diabetes and the overlapping clinical features makes diagnosing type 1 diabetes challenging. Therefore, unsurprisingly, misclassification of clinically diagnosed cases is commonly reported, with misclassification frequency increasing with onset age [28–32]. Studies defining type 1 diabetes by insulin deficiency have shown persistently retained C-peptide (in the type 2 diabetes range) in approximately 1 in 6 of those with longstanding clinically diagnosed type 1 diabetes, with low islet-antibody positivity rates and genetic susceptibility inconsistent with type 1 diabetes in these cases [28, 31]. Conversely, around 1 in 3 of those with type 1 diabetes defined by the development of severe insulin deficiency are treated without insulin at diagnosis, with 47% of these individuals still reporting type 2 diabetes at 17 years of diabetes duration [29, 30]. Findings from an analysis based on type 1 diabetes genetic risk in a large UK type 1 diabetes study are consistent with C-peptide-based studies, suggesting that two-thirds of adults with islet-autoantibody negativity who were initially diagnosed and treated as having type 1 diabetes (representing 13% of all adult-onset cases) are unlikely to have type 1 diabetes [32].

**Fig. 3:**
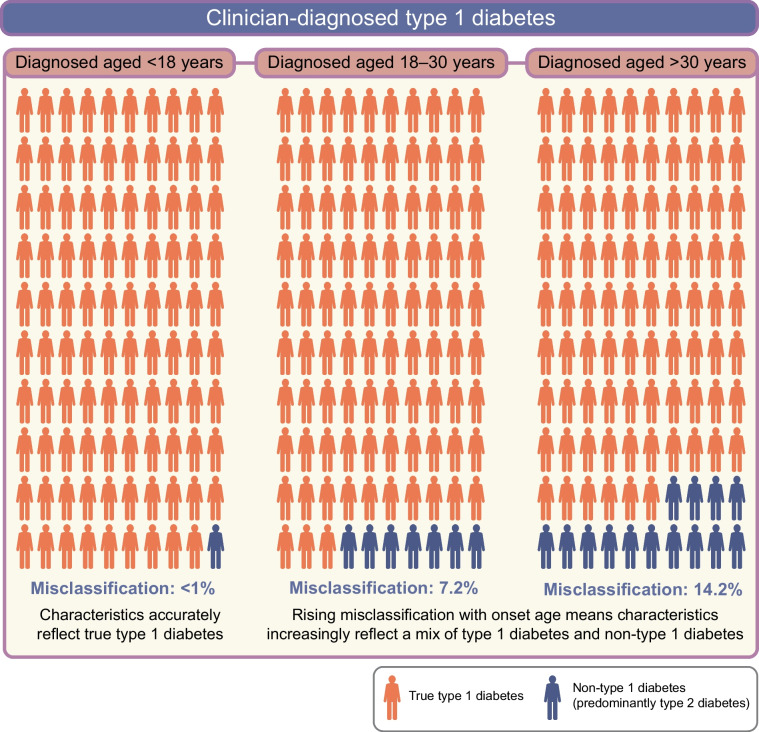
The impact of misclassification of clinician-classified type 1 diabetes on the observed clinical features of type 1 diabetes. Rates of misclassification taken from [28]. This figure is available as part of a downloadable slideset

These high levels of misclassification not only have an impact on individual patient management but will also influence the observed phenotype of type 1 diabetes in research studies. Reported characteristics of clinician-diagnosed type 1 diabetes will be influenced by misclassified non-autoimmune cases within a study cohort (Fig. 3). Of cohorts with clinically diagnosed type 1 diabetes, the proportion misclassified will increase with increasing age, thus enriching for characteristics associated with type 2 diabetes as cohort age increases, even if the true phenotype of late-onset type 1 diabetes is unaltered [4, 5, 28, 30].

## The marked changes in characteristics of clinically diagnosed type 1 diabetes with later onset may largely be explained by misclassification

In this section we evaluate the reported characteristics of adult-onset type 1 diabetes and how these features may be impacted by the definition of type 1 diabetes used.

### Clinical features of adult-onset type 1 diabetes

The features of clinician-diagnosed type 1 diabetes are observed to change with increasing onset age. Adult type 1 diabetes cohorts are typically reported to have higher BMI and less severe symptoms than children, including lower presentation HbA_1c_, and less frequent pre-diagnosis weight loss and ketoacidosis [33–36]. Conversely, when adult-onset type 1 diabetes is classified by high-specificity definitions, the impact of age on clinical features appears minimal. We recently evaluated the impact of onset age on clinical presentation in adults. Type 1 diabetes was defined by ≥2 positive autoantibodies (of GADA, IA-2A or ZnT8) irrespective of reported clinician diagnosis. Presentation features, including symptoms, ketoacidosis and blood glucose levels, were similar in those diagnosed before and after 35 years of age. However, despite similar characteristics, those diagnosed at an older age were far less likely to be admitted to hospital at diagnosis, start insulin or be diagnosed as having type 1 diabetes [37]. Consistent with this, in studies of longstanding adult-onset type 1 diabetes defined by either low C-peptide or genetically, current clinical features, including BMI, HbA_1c_, insulin dose and ketoacidosis rates, were comparable irrespective of onset age [1, 30]. These findings suggest that, when robustly defined, clinical features of type 1 diabetes are similar across adult-onset age, and the more type 2 diabetes-like phenotype often reported with later onset age may reflect increasing misclassification.

### Genetic architecture of adult-onset type 1 diabetes

In clinician-diagnosed type 1 diabetes, polygenetic predisposition decreases with increasing onset age, reflecting marked differences in HLA-associated type 1 diabetes risk [34, 38, 39] (see electronic supplementary material [ESM] Table 1). In adult cohorts with clinically diagnosed type 1 diabetes, genetic overlap with type 2 diabetes is observed, particularly with increasing onset age [40, 41]. In contrast, when a clinical diagnosis of adult-onset type 1 diabetes is confirmed by autoantibody positivity, genetic predisposition to type 1 diabetes appears unaffected by onset age but is modestly reduced relative to childhood-onset cases [32, 38]. Adults, but not children, with islet-autoantibody negativity had substantially lower type 1 diabetes genetic susceptibility than those who were islet-autoantibody positive, consistent with the presence of non-autoimmune diabetes [31, 32].

### Rates of islet autoantibodies in adult-onset type 1 diabetes

**Fig. 4:**
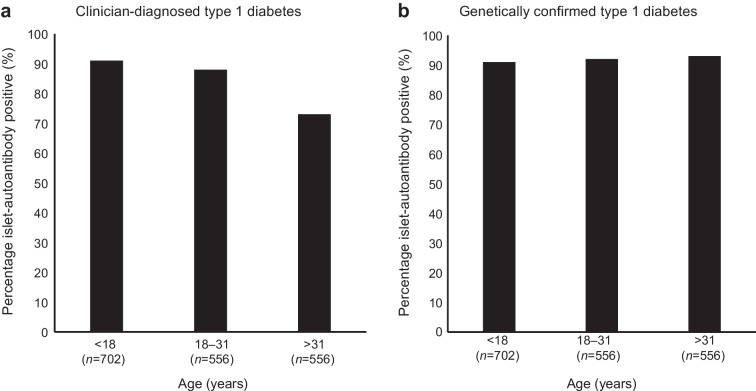
The pattern of islet autoantibodies at diagnosis in (**a**) clinician-diagnosed type 1 diabetes in children (<18 years old), young adults (18–31 years old) and older adults (>31 years old) and (**b**) after genetic adjustment for non-autoimmune diabetes. Data from [13, 32]. This figure is available as part of a downloadable slideset

A number of studies have demonstrated that in clinician-diagnosed type 1 diabetes positive islet autoantibodies are less common in adults than in children, with positivity dropping with older onset age [13, 34, 36, 42] (ESM Table 2). For example, in a recent study assessing GADA, ZnT8A and IA-2A in new-onset clinically diagnosed type 1 diabetes, children were more likely to have at least one positive islet autoantibody than adults (90% vs 82%), and the absence of islet autoantibodies was most marked in older adults, with around one-quarter (27%) of those diagnosed at >31 years of age being islet-autoantibody negative (Fig. 4) [13].

The reduction in islet-autoantibody positivity in clinically diagnosed type 1 diabetes with increasing age may be explained by higher rates of misclassification with increasing onset age. In a recent study of new-onset clinician-diagnosed type 1 diabetes, we observed a substantial reduction in genetic susceptibility to type 1 diabetes within adults who were islet-autoantibody negative, consistent with an estimated two-thirds (67%) of cases having non-autoimmune diabetes [32]. When adjusted for these genetically estimated non-autoimmune diabetes cases, islet-autoantibody-positivity rates did not change with type 1 diabetes onset age [32]. Consistent with these findings, we have separately shown that islet-autoantibody-negative clinician-diagnosed type 1 diabetes, when measured within 1 year of diagnosis, had substantially slower loss of endogenous insulin secretion (C-peptide), with many participants who were initially diagnosed and treated as type 1 diabetes being able to successfully stop insulin after being informed of the negative islet-autoantibody results [31].

A consistent finding across studies, irrespective of type 1 diabetes definition, is that when autoantibodies are present, autoantibody patterns vary with onset age. In children, multi-autoantibody-positive type 1 diabetes cases are more common than in adults [35, 36, 42, 43] and IA-2A positivity is more frequent. Moreover, a higher IA-2A titre is associated with earlier type 1 diabetes onset. Conversely, in adults, GADA is the predominant autoantibody, irrespective of ethnicity [13, 32, 35, 36, 43]. Islet autoantibodies are more persistent after diagnosis in adults than in children, which may reflect the greater prevalence of GADA and/or retained antigenic stimulus [44].

### Progression of insulin loss in adult-onset type 1 diabetes

Cross-sectional studies of clinician-diagnosed type 1 diabetes show that a higher proportion of participants with longstanding adult-onset diabetes retain measurable endogenous insulin secretion compared with childhood-onset diabetes [40, 45], with the prevalence of preserved C-peptide secretion being highest in those with older diagnosis age [40]. It is unclear if this reflects age-related differences in C-peptide loss or misclassification. In TRIALNET participants who were diagnosed with islet-autoantibody-positive type 1 diabetes up to the age of 45 years, insulin loss was largely unaffected by onset age in children, but in adult-onset cases, insulin loss was less rapid [46, 47]. In a recent analysis from a prospective study of participants with new-onset diabetes, aged 18–88 years, we defined type 1 diabetes by multi-autoantibody positivity (irrespective of clinician diagnosis) or, separately, by a clinician diagnosis confirmed by a single-positive islet autoantibody [37]. Over 2 years of follow up, C-peptide loss was not associated with onset age and did not differ by how type 1 diabetes was defined. Annual C-peptide loss was ~40% in adults (regardless of age or definition), which is comparable with the ~50% annual C-peptide loss reported in children [46–50]. This suggests that the high prevalence of preserved C-peptide reported in individuals diagnosed with type 1 diabetes as older adults may at least partly reflect increasing misclassification with increasing onset age.

### Summary: the impact of onset age on the characteristics of adult-onset type 1 diabetes

In summary, many of the changes in clinical, biomarker and genetic characteristics previously reported with increasing age of type 1 diabetes onset may be explained by the inadvertent study of mixed populations with and without diabetes with autoimmune aetiology, arising from difficulties in robustly identifying type 1 diabetes in adults. When adult type 1 diabetes is defined by methods likely to result in low misclassification rates, the impact of age on type 1 diabetes characteristics is modest. Although studies directly comparing robustly defined type 1 diabetes at all ages of onset are lacking, compared with childhood-onset, adult-onset type 1 diabetes appears to have similar rates of islet-autoantibody positivity (though both the types of autoantibodies present and titre may differ), and only modest differences in progression of beta cell failure and genetic susceptibility. One feature appearing to markedly vary with age of type 1 diabetes onset is clinician practice: despite similar clinical presentation, adults with type 1 diabetes appear to be less frequently identified or initially treated with insulin.

## Recommendations for classifying adult-onset type 1 diabetes in clinical practice

This section aims to highlight key issues for identifying adult-onset type 1 diabetes in clinical practice, to supplement recent published guidelines for diabetes classification [51–53]. See Text box for a summary of these recommendations.

### Recommendation 1

#### Clinical features are often insufficient to confirm a diagnosis of adult-onset type 1 diabetes: biomarker confirmation is needed

The high prior likelihood of non-autoimmune diabetes in adults (especially at older onset), combined with overlapping clinical features with other diabetes subtypes makes it challenging to robustly diagnose type 1 diabetes using clinical features alone [6]. Therefore, confirmatory biomarker testing should be strongly considered, especially where features suggestive of non-type 1 diabetes are present. Consistent with this, recent guidance from the EASD/ADA and the UK National Institute for Health and Care Excellence recommend routine islet-autoantibody testing in all adults who develop clinically suspected type 1 diabetes, with C-peptide testing after 3 years of duration in all those who are islet-autoantibody negative [51–54]. In this high-prior-prevalence setting, the finding of positive islet autoantibodies, if assessed using modern high-specificity assays, will usually confirm type 1 diabetes [28, 31].

### Recommendation 2

#### Strategies for managing autoantibody-negative clinically suspected type 1 diabetes: consider alternative diagnosis and potential cessation of insulin therapy

Clinicians are often taught that negative islet autoantibodies are a poor rule-out test for type 1 diabetes. This assertion is based on findings from childhood onset where the high prior likelihood of type 1 diabetes means most of those living with diabetes will have type 1 diabetes, irrespective of islet-autoantibody status [32]. In adults, the high prior prevalence of type 2 diabetes means negative islet autoantibodies (if measured close to diagnosis) will be strongly suggestive of diabetes with a non-autoimmune aetiology (type 2 diabetes or MODY), with research suggesting that the majority of islet-autoantibody-negative adults clinically thought to have type 1 diabetes are not likely to have this condition and that many individuals within this group can successfully and safely stop insulin treatment [31, 32]. Therefore, in adults, negative islet autoantibodies should prompt consideration of other diabetes subtypes, and potentially the trial of non-insulin treatment and insulin cessation [16, 28, 31, 53]. C-peptide tests should always be undertaken before considering insulin withdrawal (interpretation of the test is discussed under ‘Recommendation 5’ below). Importantly, endogenous insulin secretion may be maintained at type 1 diabetes diagnosis (particularly in individuals with obesity), but rapidly fall [14]. Therefore, any attempt of insulin withdrawal needs very careful patient education and ongoing monitoring, even if initially successful. Where trial of non-insulin therapy is not undertaken and MODY is excluded our practice, in line with international guidance, is to keep the subtype under review and undertake serial C-peptide measurement [51–54].

### Recommendation 3

#### Islet-autoantibody testing should not be routinely undertaken in those with apparent type 2 diabetes

Where the likelihood of type 1 diabetes is low (for example, in those with a classical type 2 diabetes presentation) a single-positive islet-autoantibody finding will not confirm diabetes with an autoimmune aetiology [4, 5] and, at present, international guidelines do not recommend routine testing in this setting. In our opinion, routine testing in those with apparent type 2 diabetes is likely to lead to high levels of false-positive findings and potential patient harm and, therefore, should not be undertaken. However we acknowledge this should be weighed against the harms of missed type 1 diabetes and challenges of identifying type 1 diabetes based on clinical features alone. Therefore, testing with appropriately cautious interpretation of results may be a valid clinical strategy. The impact and cost effectiveness of routine islet-autoantibody testing in this situation is an important area for further research.

If islet autoantibodies have been assessed in this context and a single-positive islet autoantibody is detected, our practice is to continue management as type 2 diabetes, with additional education and careful monitoring, and revise diagnosis and treatment if there is rapid progression (see ‘Recommendation 4’ below).

Clinical diagnosis is not systematic and prior probability is hard to accurately determine by an individual clinician. Therefore, an alternative approach to guide islet-autoantibody testing and the interpretation of results is to use prediction models that combine clinical features and (where required) islet autoantibodies and/or other classification biomarkers. Models using combined clinical features with or without islet autoantibodies and a type 1 diabetes genetic risk score (T1D-GRS) have been developed and validated in European populations [55–57], but have not yet been prospectively validated at diagnosis or in individuals with non-white ethnicity. Separate models have been developed in a Chinese population, showing high performance [58]. The presence of multiple positive islet autoantibodies, assessed using modern high-specificity assays, is associated with very high specificity and, in most cases, will confirm type 1 diabetes even if the pre-test likelihood of type 1 diabetes is low [42, 59]. A caution for clinical interpretation of islet-autoantibody testing is that specificity can vary widely despite recent improvements in assays, thus, data from appropriate control populations is crucial for interpretation [60].

### Recommendation 4

#### Requirement of insulin within 3 years of diagnosis of apparent type 2 diabetes should trigger assessment with classification biomarkers

A key clinical feature for the recognition of adults who are initially diagnosed as type 2 diabetes who have misdiagnosed type 1 diabetes is that insulin is rapidly required to control hyperglycaemia. Many of those developing type 1 diabetes in later life will initially be classified and treated as having type 2 diabetes. Recent studies of adult-onset type 1 diabetes defined using genetic methods or low C-peptide values suggest that the vast majority of those with unrecognised type 1 diabetes who are initially diagnosed as having type 2 diabetes progress to insulin with 3 years of diagnosis, with most requiring insulin within 1 year [1, 30]. In a UK cohort, approximately 25% of those aged >30 years who were initially diagnosed and treated as having type 2 diabetes but progressed to insulin treatment within 3 years developed near absolute insulin deficiency (non-fasting C-peptide <200 pmol/l), and these participants had genetic and islet-autoantibody characteristics associated with type 1 diabetes [30]. This patient group should therefore undergo islet-autoantibody or (if long duration) C-peptide testing. The high probability of type 1 diabetes in this group means that a positive islet autoantibody is mathematically likely to confirm diabetes of autoimmune aetiology.

### Recommendation 5

#### In longstanding diabetes (>3 years’ duration), measuring C-peptide is likely to confirm treatment requirements

Current guidelines recommend C-peptide testing after 3 years’ diabetes duration where there is diagnostic uncertainty [51–54]. The glycaemic treatment, monitoring and education requirements of type 1 diabetes is driven principally by the development of severe endogenous insulin deficiency [14, 61, 62]. In longstanding diabetes, it is therefore appropriate that C-peptide assessment is the initial test for guiding clinical management and (outside of monogenic diabetes diagnosis) it is currently unclear if islet autoantibodies have utility over and above C-peptide. Low C-peptide (approximately <200–300 pmol/l) confirms the treatment requirements of type 1 diabetes, including insulin requirement [14, 61, 63]. Conversely, persistent high C-peptide levels (approximately >600 pmol/l) indicate substantial retained insulin secretion consistent with the treatment requirements and response of type 2 diabetes [14]. Many patients diagnosed with type 1 diabetes who maintain C-peptide in this range can improve blood glucose levels with adjuvant (type 2 diabetes-associated) agents and/or discontinue insulin [28, 31]. Persistent intermediate values are consistent with either type 1 diabetes or MODY but can occur in longstanding type 2 diabetes or in individuals with type 2 diabetes and low BMI. While it is likely that some individuals in this group will not require insulin and may benefit from adjuvant (type 2 diabetes-associated) agents, clinical studies are limited [14].

Recent evidence suggests that routine C-peptide testing in those with clinically diagnosed type 1 diabetes may be cost effective. In a UK centre, routine C-peptide testing of all adult-onset type 1 diabetes resulted in 11% of individuals being re-classified, with~25% of these individuals discontinuing insulin therapy. This strategy was believed to be cost saving based on saved treatment costs alone [28].

## Recommendations for researchers

The difficulty of diagnosing type 1 diabetes in adults means misclassification in this age group is common, with type 1 diabetes cohorts who are defined by clinical diagnosis or a single-positive islet autoantibody alone being likely to include many misclassified individuals. Therefore, when interpreting studies on adult-onset autoimmune diabetes, we recommend careful consideration as to whether inadvertent inclusion of diabetes with a non-autoimmune aetiology may explain study findings. To understand the characteristics of type 1 diabetes in adults it is essential that studies define cases using methods that minimise misclassification. For many research questions, a clinical diagnosis confirmed by (subsequent) positive islet autoantibodies will be appropriate. Where islet antibodies are measured, it is critical that test specificity is reported. For researching type 1 diabetes irrespective of clinical diagnosis, very high-specificity approaches are needed, such as confirming multiple positive islet autoantibodies [37] or near absolute endogenous insulin deficiency [30] and using recently developed genotype-based methods [1, 64]. The optimum method depends on the research question; for example, defining type 1 diabetes by low endogenous insulin precludes studying disease severity.

## Conclusion

Diagnosing type 1 diabetes in adults is difficult as presentation can overlap with type 2 diabetes, which is vastly more common than type 1 diabetes with increasing onset age. Many of the reported age-related changes in type 1 diabetes phenotype may be explained by increasing inclusion of misclassified non-autoimmune diabetes with increased age at diagnosis. For clinicians, biomarker investigation is essential both to confirm a clinical diagnosis of adult-onset type 1 diabetes, and to determine subtype in those initially diagnosed as having type 2 diabetes but who have rapid disease progression. For researchers, the use of high-specificity approaches to define type 1 diabetes in adults is critical to understanding the phenotype of adult-onset autoimmune aetiology diabetes.

### Supplementary Information

Below is the link to the electronic supplementary material.

Slideset of figures (PPTX 424 KB)

ESM Tables (PDF 238 KB)

## Abbreviations

LADA: Latent autoimmune diabetes of adults
PPV: Positive predictive value
