# Supplementary material for: The challenges of identifying and studying type 1 diabetes in adults
Source: Diabetologia. 2023 Sep 20;66(12):2200–12. doi: 10.1007/s00125-023-06004-4 (PMC10628058; doi:10.1007/s00125-023-06004-4)
Supplement: Supplementary file 1 — Slideset of figures (PPTX 424 KB) [file 125_2023_6004_MOESM1_ESM.pptx]

## Slide 1
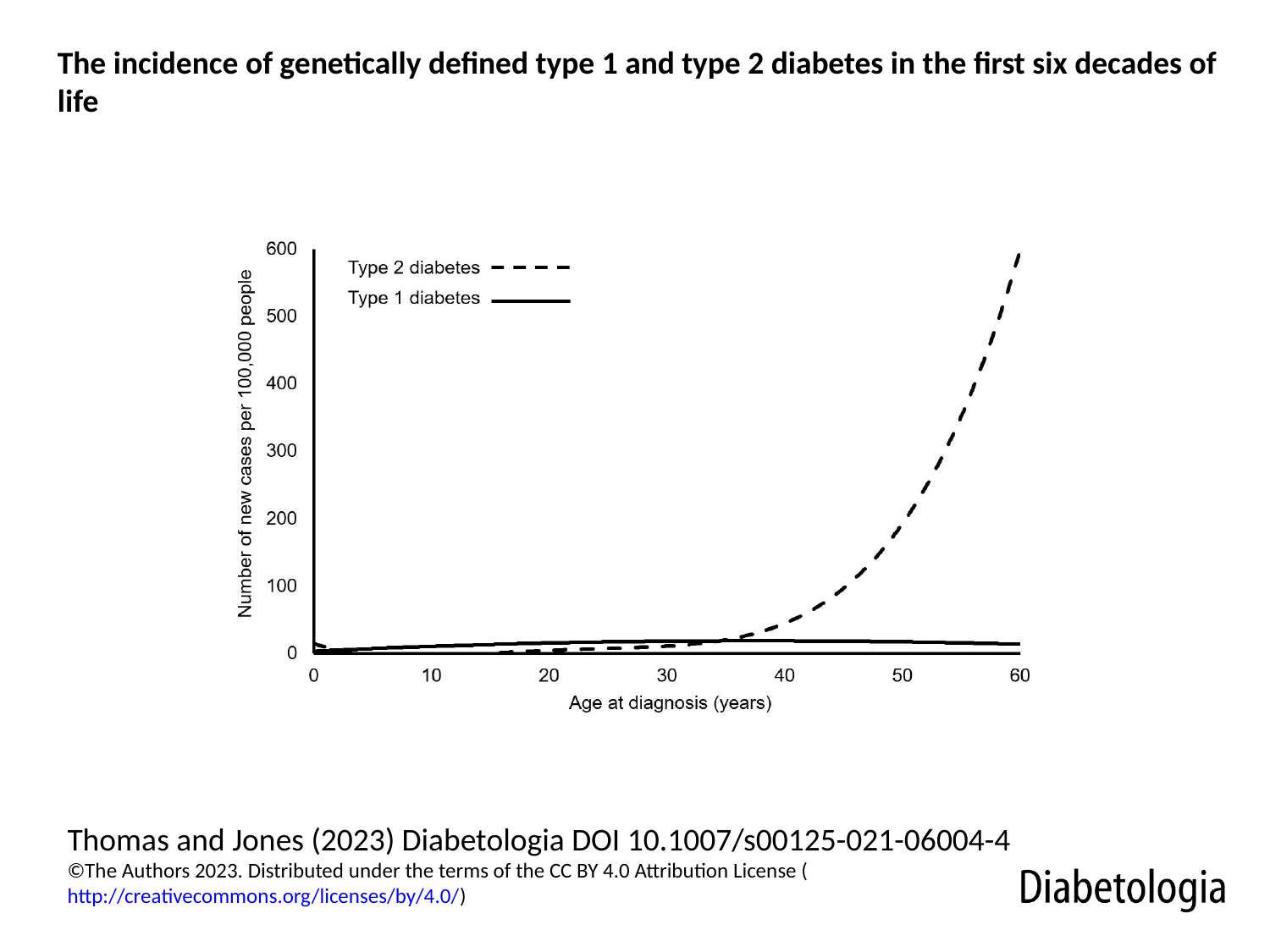

The incidence of genetically defined type 1 and type 2 diabetes in the first six decades of life
Thomas and Jones (2023) Diabetologia DOI 10.1007/s00125-021-06004-4
©The Authors 2023. Distributed under the terms of the CC BY 4.0 Attribution License (http://creativecommons.org/licenses/by/4.0/)

## Slide 2
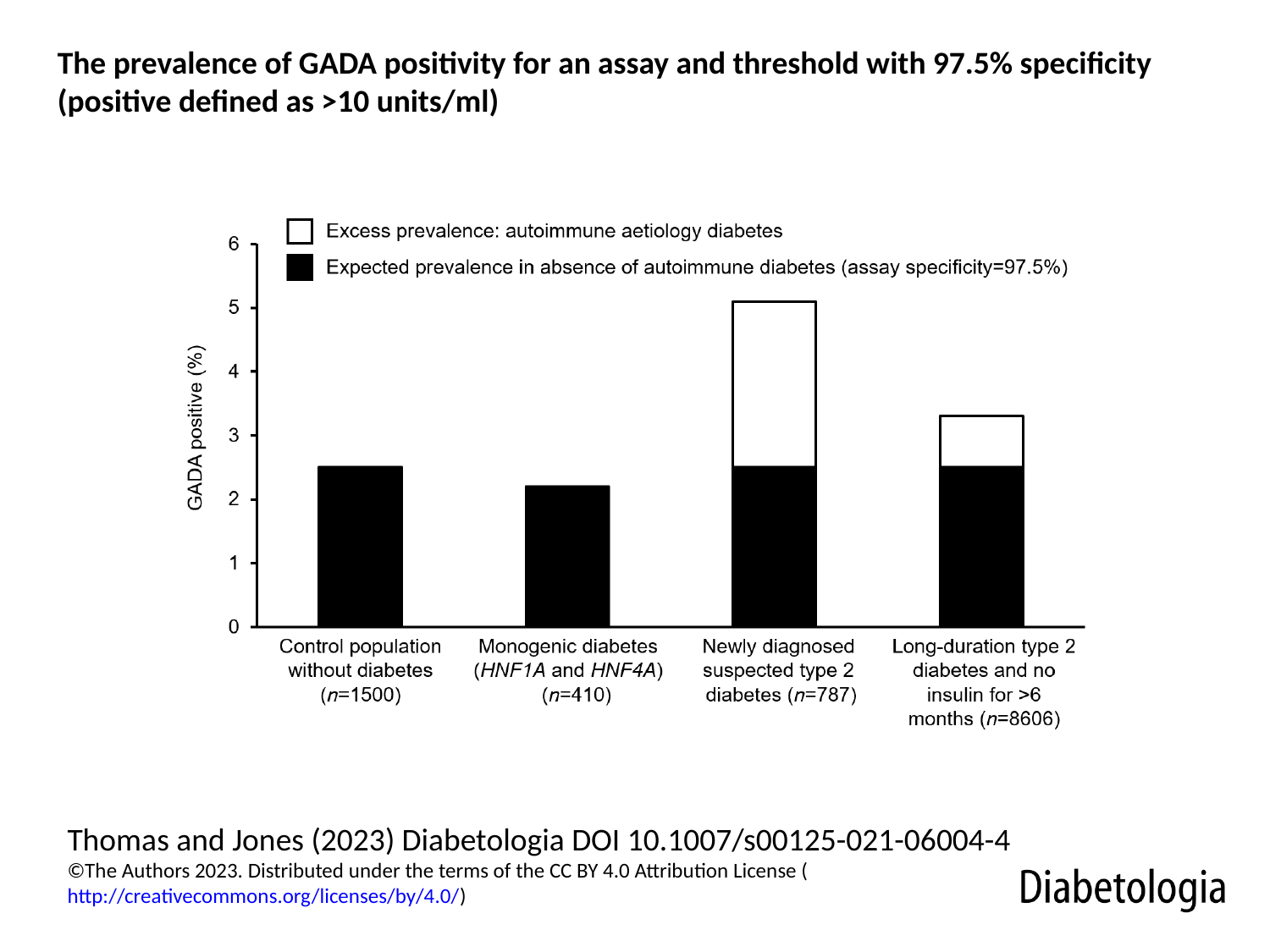

The prevalence of GADA positivity for an assay and threshold with 97.5% specificity (positive defined as >10 units/ml)
Thomas and Jones (2023) Diabetologia DOI 10.1007/s00125-021-06004-4
©The Authors 2023. Distributed under the terms of the CC BY 4.0 Attribution License (http://creativecommons.org/licenses/by/4.0/)

## Slide 3
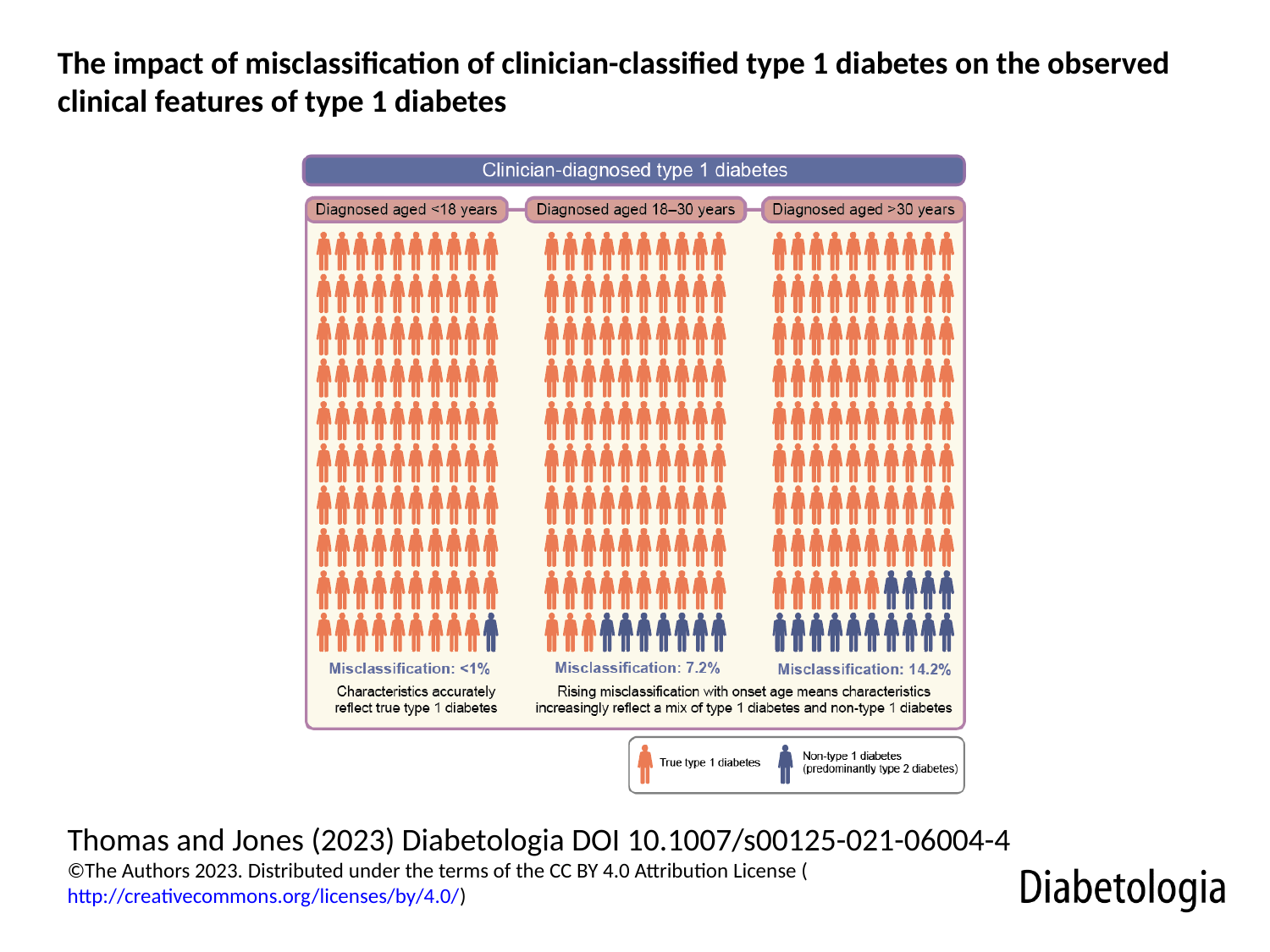

The impact of misclassification of clinician-classified type 1 diabetes on the observed clinical features of type 1 diabetes
Thomas and Jones (2023) Diabetologia DOI 10.1007/s00125-021-06004-4
©The Authors 2023. Distributed under the terms of the CC BY 4.0 Attribution License (http://creativecommons.org/licenses/by/4.0/)

## Slide 4
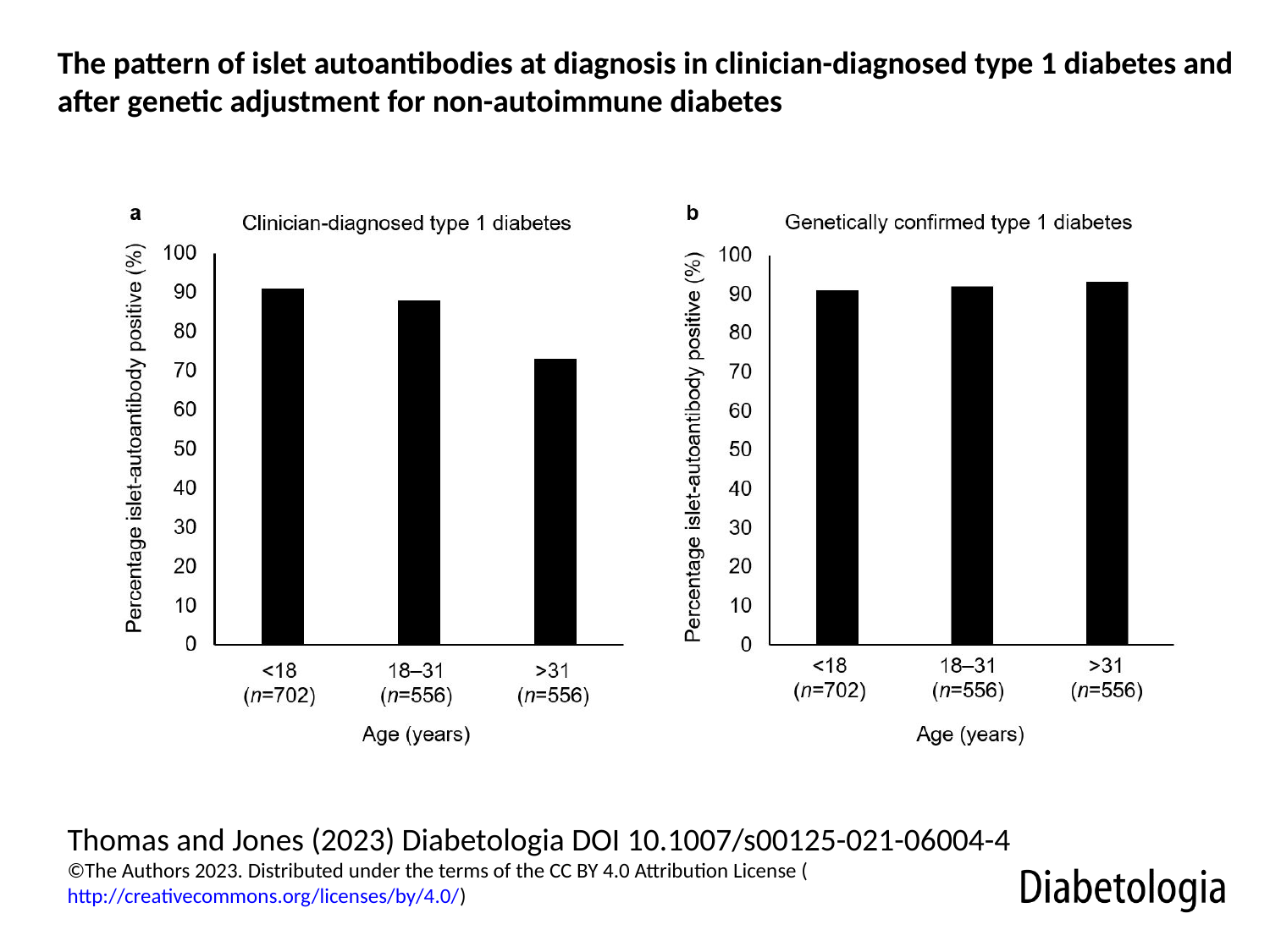

The pattern of islet autoantibodies at diagnosis in clinician-diagnosed type 1 diabetes and after genetic adjustment for non-autoimmune diabetes
Thomas and Jones (2023) Diabetologia DOI 10.1007/s00125-021-06004-4
©The Authors 2023. Distributed under the terms of the CC BY 4.0 Attribution License (http://creativecommons.org/licenses/by/4.0/)
