# Supplementary material for: The challenges of identifying and studying type 1 diabetes in adults
Source: Diabetologia. 2023 Sep 20;66(12):2200–12. doi: 10.1007/s00125-023-06004-4 (PMC10628058; doi:10.1007/s00125-023-06004-4)
Supplement: Supplementary file 2 — ESM Tables (PDF 238 KB) [file 125_2023_6004_MOESM2_ESM.pdf]

ESM Table 1: *Type 1 diabetes studies evaluating the impact of age at diagnosis on the genetic risk for type 1 diabetes. The highest risk HLA genotype (DR3-DQ2/DR4-DQ8) and protective haplotype (DR15-DQ6) are reported. No upper age was reported if not stated. Where statistical comparisons between age groups were not stated in the original study Chi-squared test was used to compare age groups by HLA status. ^ p value reported for change in odds ratio with increasing onset age.*

		**Ages studied (n)**		**Genetic associations with increasing age**			
**Study (reference)**	**How type 1 diabetes defined**	**Younger age group**	**Older age group**	**DR3-DQ2/DR4-DQ8 (Younger vs Older)**	**p**	**DR15-DQ6 (Younger vs Older)**	**p**
**Karjalainen et.al (1989) (1)**	Clinician Diagnosed	≤18 years (82)	>18 to 56 years (44)	37% (26 to 47%) vs 13% (2 to 23%)	0.01	Not Reported	N/A
**Caillat-Zucman et.al (1992) (2)**	Clinician Diagnosed	<15 years (112)	≥15 years (290)	38% (29 to 46%) vs 18% (14 to 22%)	<0.0001	3% (0 to 6%) vs 5% (2 to 7%)	0.4
		15-30 years (183)	>30 years (107)	24% (18 to 30%) vs 10% (5 to 16%)	<0.01	Not Reported	N/A
**Vandewalle et.al (1993) (3)**	Clinician Diagnosed	≤19 years (126)	20 to 39 years (153)	40% (31 to 48%) vs 20% (14 to 26%)	<0.001	Not Reported	N/A
**Tait et.al (1995) (4)**	Clinician Diagnosed & C-peptide <0.1nm	≤20 years (115)	>21 years (79)	48% (39% to 57%) vs 29% (19 to 39%)	<0.01	3% (0 to 8%) vs 8% (0 to 18%)	0.2
**Graham el.al (1999) (5)**	Clinician Diagnosed	≤20 years (566)	≥20 to 34 years (264)	33% (30 to 37%) vs 24% (19 to 29%)	0.02^	1% (0 to 2%) vs 3% (2 to 7%)	<0.001^
**Sabbah et.al (2000) (6)**	Clinician Diagnosed	<20years (188)	≥20 to 62 years (59)	23% (17 to 29%) vs 8% (1 to 16%)	0.01	2% (0 to 3%) vs 10% (2 to 18%)	<0.01
**Petrone et.al (2005) (7)**	Clinician Diagnosed	<15 years (650)	≥15 to 49 years (221)	24% (21 to 27%) vs 14% (9 to 19%)	<0.01	Not Reported	N/A
**Howson et.al (2011) (8)**	Clinician Diagnosed & ≥1 autoantibody	3 to ≤21 years (325)	>21 to 89 years (944)	23% (18 to 27%) vs 20% (17 to 22%)	0.2	2% (0 to 3%) vs 4% (3 to 6%)	0.02
**Thomas et.al (2022) (9)**	Clinician Diagnosed & ≥1 autoantibody	<18 years (n=642)	≥18 (n=894)	28% (25 to 32%) vs 19% (16 to 22%)	<0.0001	2% (1 to 3%) vs 3% (2 to 4%)	0.1

ESM Table 2*. Studies evaluating the impact of age at diagnosis on percentage of type 1 diabetes cases islet autoantibody positive. ^ Autoantibodies tested within 1 year of diagnosis, ^^ Autoantibodies tested within 2 years of diagnosis. No upper age was reported if not stated. Where statistical comparisons between age groups were not stated in the original study Chi-squared test was used to compare autoantibody status between age groups.*

			**Ages studied**			**Antibody positive**		
**Study**	**Type 1 diabetes definition**	**Autoantibodies measured**	**Younger age group**	**Older age group**	**autoantibodies reported**	**Children**	**Adults**	**P**
**Lohmann et.al (1997) (10)^^**	Clinician Diagnosed	ICA, GADA, IA-2A	12-40 years (22)	>40-67 years (24)	≥1 Ab +ve<br>GADA	83% (67 to 98)<br>74% (60 to 95)	58% (39 to 78)<br>29% (11 to 47)	0.07<br><0.01
**Sabbah et.al (2000) (6) ^**	Clinician Diagnosed	ICA, IAA, GADA, IA-2A	<20 years (252)	≥20-62 years (100)	≥1 Ab +ve<br>GADA<br>≥2 Ab +ve	96% (94 to 98)<br>68% (62 to 74)<br>70% (64 to 76)	70% (61 to 79)<br>51% (41 to 61)<br>34% (25 to 43)	<0.0001<br><0.01<br><0.001
**Vermeulen et.al (2011) (11) ^**	Clinician Diagnosed	GADA, IA-2A, ZNT8	≤19 years (393)	>19-40 years (262)	≥1 Ab +ve<br>≥2 Ab +ve	94% (92 to 97)<br>73% (68 to 77)	79% (74 to 84)<br>53% (47 to 59)	<0.0001<br><0.0001
**Bravis et.al (2018) (12) ^**	Clinician Diagnosed	GADA, IA-2A, ZNT8	<17 years (676)	≥17 years (1102)	≥1 Ab +ve	90% (88 to 92)	82% (80 to 84)	<0.0001
**Rogowicz-Frontczak et.al (2018) (13) ^**	Clinician Diagnosed	ICA, GADA, IA-2A, ZNT8	<35 years (66)	≥35 years (53)	≥1 Ab +ve<br>GADA<br>≥2 Ab +ve	92% (86 to 99)<br>82% (72 to 91)<br>88% (80 to 96)	85% (75 to 95)<br>77% (66 to 89)<br>55% (41 to 68)	0.2<br>0.5<br><0.0001
**Niechcial et.al (2018) (14) ^**	Clinician Diagnosed	GADA, IA-2A, ZNT8	<18 years (218)	≥18 years (149)	≥1 Ab +ve<br>GADA<br>≥2 Ab +ve	95% (92 to 98)<br>87% (83 to 92)<br>70% (64 to 76)	81% (75 to 87)<br>49% (41 to 57)<br>74% (67 to 81)	<0.0001<br><0.0001<br>0.3
**Luo et.al (2020) (15) ^^**	Clinician Diagnosed	GADA, IA-2A, ZNT8	≤20 years (340)	>20-70 years (411)	≥1 Ab +ve<br>GADA<br>≥2 Ab +ve	76% (71 to 82)<br>61% (54 to 67)<br>39% (33 o 46)	63% (57 to 69)<br>57% (51 to 63)<br>24% (19 to 29)	<0.01<br>0.4<br><0.001
**Thomas et.al (2022) (9) ^**	Genetic stratification	GADA, IA-2A, ZNT8	18-31 years (n=576)	>31 (n=556)	≥1 Ab +ve	92% (89 to 94)	93% (91 to 95)	0.4

ESM References

1. Karjalainen J, Knip M, Hyoty H, Leinikki P, Ilonen J, Kaar ML, et al. Relationship between serum insulin autoantibodies, islet cell antibodies and Coxsackie-B4 and mumps virus-specific antibodies at the clinical manifestation of type 1 (insulin-dependent) diabetes. Diabetologia. 1988;31(3):146-52.
2. Caillat-Zucman S, Garchon HJ, Timsit J, Assan R, Boitard C, Djilali-Saiah I, et al. Age-dependent HLA genetic heterogeneity of type 1 insulin-dependent diabetes mellitus. J Clin Invest. 1992;90(6):2242-50.
3. Vandewalle CL, Decraene T, Schuit FC, De Leeuw IH, Pipeleers DG, Gorus FK. Insulin autoantibodies and high titre islet cell antibodies are preferentially associated with the HLA DQA1*0301-DQB1*0302 haplotype at clinical type 1 (insulin-dependent) diabetes mellitus before age 10 years, but not at onset between age 10 and 40 years. The Belgian Diabetes Registry. Diabetologia. 1993;36(11):1155-62.
4. Tait BD, Harrison LC, Drummond BP, Stewart V, Varney MD, Honeyman MC. HLA antigens and age at diagnosis of insulin-dependent diabetes mellitus. Hum Immunol. 1995;42(2):116-22.
5. Graham J, Kockum I, Sanjeevi CB, Landin-Olsson M, Nystrom L, Sundkvist G, et al. Negative association between type 1 diabetes and HLA DQB1*0602-DQA1*0102 is attenuated with age at onset. Swedish Childhood Diabetes Study Group. Eur J Immunogenet. 1999;26(2-3):117-27.
6. Sabbah E, Savola K, Ebeling T, Kulmala P, Vahasalo P, Ilonen J, et al. Genetic, autoimmune, and clinical characteristics of childhood- and adult-onset type 1 diabetes. Diabetes Care. 2000;23(9):1326-32.
7. Petrone A, Galgani A, Spoletini M, Alemanno I, Di Cola S, Bassotti G, et al. Residual insulin secretion at diagnosis of type 1 diabetes is independently associated with both, age of onset and HLA genotype. Diabetes Metab Res Rev. 2005;21(3):271-5.
8. Howson JMM, Rosinger S, Smyth DJ, Boehm BO, Todd JA, Grp A-ES. Genetic Analysis of Adult-Onset Autoimmune Diabetes. Diabetes. 2011;60(10):2645-53.
9. Thomas NJ, Hill AV, Dayan CM, Oram RA, McDonald TJ, Shields BM, et al. Age of Diagnosis Does Not Alter the Presentation or Progression of Robustly Defined Adult-Onset Type 1 Diabetes. Diabetes Care. 2023.
10. Lohmann T, Sessler J, Verlohren HJ, Schroder S, Rotger J, Dahn K, et al. Distinct genetic and immunological features in patients with onset of IDDM before and after age 40. Diabetes Care. 1997;20(4):524-9.
11. Vermeulen I, Weets I, Asanghanwa M, Ruige J, Van Gaal L, Mathieu C, et al. Contribution of antibodies against IA-2beta and zinc transporter 8 to classification of diabetes diagnosed under 40 years of age. Diabetes Care. 2011;34(8):1760-5.
12. Bravis V, Kaur A, Walkey HC, Godsland IF, Misra S, Bingley PJ, et al. Relationship between islet autoantibody status and the clinical characteristics of children and adults with incident type 1 diabetes in a UK cohort. BMJ Open. 2018;8(4):e020904.
13. Rogowicz-Frontczak A, Pilacinski S, Wyka K, Wierusz-Wysocka B, Zozulinska-Ziolkiewicz D. Zinc transporter 8 autoantibodies (ZnT8-ab) are associated with higher prevalence of multiple diabetes-related autoantibodies in adults with type 1 diabetes. Diabetes Res Clin Pract. 2018;146:313-20.
14. Niechciał E, Rogowicz-Frontczak A, Piłaciński S, Fichna M, Skowrońska B, Fichna P, et al. Autoantibodies against zinc transporter 8 are related to age and metabolic state in patients with newly diagnosed autoimmune diabetes. Acta Diabetol. 2018;55(3):287-94.
15. Luo S, Li X, Huang G, Xie Z, Xiang Y, Dai Z, et al. Distinct two different ages associated with clinical profiles of acute onset type 1 diabetes in Chinese patients. Diabetes Metab Res Rev. 2020;36(2):e3209.
